# Correlation between mumps and meteorological factors in Xiamen City, China: A modelling study

**DOI:** 10.1016/j.idm.2022.04.004

**Published:** 2022-04-24

**Authors:** Jie-feng Huang, Ze-yu Zhao, Wen-kui Lu, Jia Rui, Bin Deng, Wei-kang Liu, Tian-long Yang, Zhuo-yang Li, Pei-hua Li, Chan Liu, Li Luo, Bin Zhao, Yi-fang Wang, Qun Li, Ming-zhai Wang, Tianmu Chen

**Affiliations:** a State Key Laboratory of Molecular Vaccinology and Molecular Diagnostics, School of Public Health, Xiamen University, Xiamen City, Fujian Province, China; b Xiamen Center for Disease Control and Prevention, Xiamen City, Fujian Province, China; c Xiang'an Hospital of Xiamen University, Xiamen City, Fujian Province, China; d Public Health Emergency Center, Chinese Center for Disease Control and Prevention, Changping District, Beijing City, China

**Keywords:** Mumps, Transmissibility, Meteorological factors, Correlation, Time-dependent reproduction number

## Abstract

**Objective:**

Mumps is a seasonal infectious disease, always occurring in winter and spring. In this study, we aim to analyze its epidemiological characteristics, transmissibility, and its correlation with meteorological variables.

**Method:**

A seasonal *Susceptible–Exposed–Infectious/Asymptomatic–Recovered* model and a next-generation matrix method were applied to estimate the *time-dependent reproduction number* (*R*_*t*_).

**Results:**

The seasonal double peak of annual incidence was mainly in May to July and November to December. There was high transmission at the median of *R*_*t*_ = 1.091 (ranged: 0 to 4.393). *R*_*t*_ was seasonally distributed mainly from February to April and from September to November. Correlations were found between temperature (Pearson correlation coefficient [*r*] ranged: from 0.101 to 0.115), average relative humidity (*r* = 0.070), average local pressure (*r* = -0.066), and the number of new cases. In addition, average local pressure (*r* = 0.188), average wind speed (*r* = 0.111), air temperature (*r* ranged: -0.128 to -0.150), average relative humidity (*r* = -0.203) and sunshine duration (*r* = -0.075) were all correlated with *R*_*t*_.

**Conclusion:**

A relatively high level of transmissibility has been found in Xiamen City, leading to a continuous epidemic of mumps. Meteorological factors, especially air temperature and relative humidity, may be more closely associated with mumps than other factors.

## 1 Introduction

Mumps is a common viral disease in children and is an acute respiratory infection caused by the mumps virus. Mumps is highly contagious in humans. In China, mumps is classified as a Class C notifiable infectious disease (Court, 2006). Although some measures have been taken to prevent mumps, including vaccination (Hviid et al., 2008), the epidemic remains severe due to genetic mutation in the mumps virus as well as the large and mobile population in China. The mean incidence rate in Xiamen was 21.1/100,000 from 2005 to 2017 (Li et al., 2018), which is the higher incidence rate within Fujian Province and close to the national average (21.44/100,000) (Jiang et al., 2019). Also, as a coastal city in the south of China, Xiamen has a seasonal climate.

In China, the peak number of mumps cases shows distinct characteristics. The peak periods from 2007 to 2016 in Jining were from April to July and December to January (Zhang, Zhou, et al., 2019). The peak incidence of mumps in Hefei is from April to June (Wu et al., 2020). Guangzhou has only one peak, from May to July (Lu et al., 2020). The different seasonal characteristics of mumps throughout the country suggest that mumps may be affected by seasonal climate. In Beijing, Yu et al. used a *Seasonal Autoregressive Integrated Moving Average* (*SARIMA*) model and found that average temperature, relative humidity, vapor pressure, rainfall, and wind speed were related to the incidence of mumps (Hao et al., 2019). A *Distributed Hysteresis Nonlinear Model* (*DLNM*) was used in Fujian and precipitation, atmospheric pressure and relative humidity were found to be positively correlated with mumps (Hu et al., 2018). In a study of 10 cities including Guangxi and South China, Qi et al. used the Poisson regression model to observe a nonlinear relationship between average temperature, wind speed, and the incidence of mumps (Yu, Yang, Yu, et al., 2018). Most studies focused on the effect of meteorological variables on the onset of mumps but ignored the correlation between meteorological factors and mumps transmissibility.

Based on the occurrence, development and environmental changes of diseases, the dynamics model of infectious diseases analyzes the causes and key factors of epidemics by mathematical modeling, which provides a theoretical basis for infectious disease prevention and control decisions. In a study (Qu et al., 2017), a *Susceptible-Vaccinated-Exposed-Mild infectious-Severe infectious-Hospitalized-Recovered* (*SVEILHR*) model with periodic transmission rates was built to discuss the effect of vaccine coverage on the spread of mumps. However, this study used the basic regeneration number and did not consider the impact of meteorological. Zha et al. used *Autoregressive Integrated Moving Average* (*ARIMA*) and *Autoregressive Integrated Moving Average model with exogenous input variables* (*ARIMAX*) models with meteorological factors to simulate and predict the incidence of mumps in China (Zha et al., 2020). Two *Susceptible-Infectious-Recovered/Removed* (*SIR*) models were used in 2019 to explore the optimal allocation of vaccines during a mumps outbreak (Chernov et al., 2020). Only a few studies have analyzed the incidence and transmissibility of mumps using transmission dynamic models in which the role of meteorological factors in the transmission of mumps has been explored.

In this study, the *Susceptible-Exposed-Infectious-Asymptomatic-Recovered/Removed* (*SEIAR*) model was developed to analyze the prevalence of mumps in Xiamen City, China from 2011 to 2020, and Pearson's analysis was used to correlate the incidence of mumps with meteorology and the relationship between transmissibility and meteorological factors from 2015 to 2020.

## 2 Materials and methods

### 2.1 Data sources

Using the mumps report data set established by the Xiamen Center for Disease Control and Prevention from January 2011 to December 2020, we determined the onset date, age, and gender of each case. The data on birth rate, mortality rate, and the total population were obtained from the Xiamen Statistical Yearbook. Meteorological data (air temperature, precipitation, etc.) were obtained from the Xiamen Meteorological Bureau.

### 2.2 Transmission model

According to our previous studies, the model divided the total population into five categories: *Susceptible* (*S*), *Exposed* (*E*), *Infectious* (*I*), *Asymptomatic* (*A*), and *Recovered/Removed* (*R*). The model was based on the following assumptions:

a) Individuals (including *A* and *I*) are infectious to some degree. Susceptible individuals are infected upon contact with individuals *A* and *I.* We define the transmission relative rate as *β* and the transmissibility of *A* is *κ* (0 ≤ *κ* ≤ 1) times than *I.*
b) The proportion of asymptomatic cases occupying the sum of symptomatic and asymptomatic cases is *p.* After an incubation period (1/*ω*) and a latency period (1/*ω′*), the number of people who become symptomatic and asymptomatic cases from exposed individuals at *t* time is *pω′E* and (1-*p*)*ωE*, respectively. The model assumes that the incubation period is equal to the latent period.
c) Infected and asymptomatic individuals were transferred to recovered/removed after the 1/*γ* and 1/*γ′* infection phases, respectively.
d) The natural birth rate (*μ*) and death rate (*m*) of the population are considered in the model. *N* represents the total population.

**Fig. 1:**
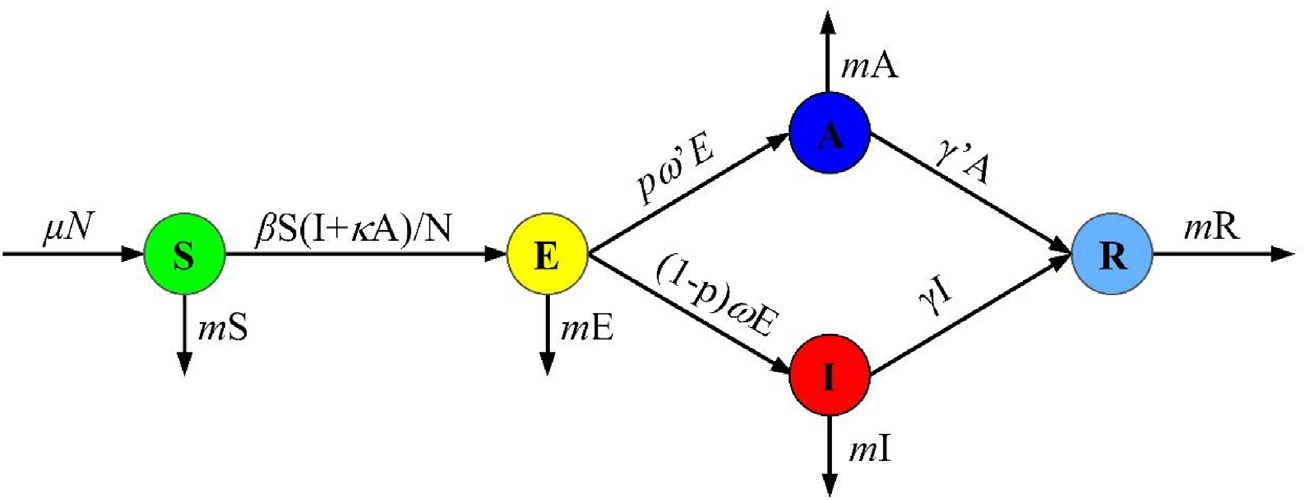
*SEIAR* model for mumps in Xiamen City.

The model is shown in Fig. 1.

The mode was expressed as follows:

$$\frac{dS}{dt}=\mu N-\beta S\left(I+\kappa A\right)/N-mS$$

$$\frac{dE}{dt}=\beta S\left(I+\kappa A\right)/N-p\omega^{\prime }E-\left(1-p\right)\omega E-mE$$

$$\frac{dI}{dt}=\left(1-p\right)\omega E-\gamma I-mI$$

$$\frac{dA}{dt}=p\omega^{\text{′}}E-\gamma^{\text{′}}A-mA$$

$$\frac{dR}{dt}=\gamma I+\gamma^{\prime }A-mR$$

N=S+E+I+A+R

The left side of the equation represents the instantaneous rate of change of *S*, *E*, *I*, *A*, and *R* in the chamber at the time *t*.

### 2.3 Seasonality simulation

In this study, we considered the seasonality of mumps in the model. According to the *SEIAR* model, we set the parameter *β* changed with a trigonometric function as follows:

$$\beta =\beta_{1}\left[1+\sin\left(time-x\right)*2*\pi /T\right]$$

$\beta_{1}$ represents the relative rate of transmission, x refers to the constant adjustment of time position, and *T* refers to the period baseline of the seasonal cycle.

### 2.4 Estimation of parameters

There are nine parameters involved in the *SEIAR* model of mumps, which are transmission relative rate *β,* relative transmissibility rate of asymptomatic to symptomatic individuals *κ*, proportion of the asymptomatic *p*, incubation relative rate *ω*, latent relative rate *ω*′, removed rate of the infectious *γ*, removed rate of the asymptomatic, birth rate *μ* and natural mortality rate *m*. Studies have shown that the incubation period of mumps is 12 – 28 days (Czumbel et al., 2018). In this study, the value was 12 days based on the available research results of the group and the actual situation in Xiamen. Assuming the incubation period and the latent period are the same (Chen et al., 2020), *ω* = *ω'* = 0.08333. Referring to the existing research (Wu et al., 2020), the proportion of recessive mumps infection was 15% – 27%, setting the value *p* = 0.2. The recovery period of the mumps is 5 – 25 days. Combined the previous study (Pan, Huang, & Chen, 2012) and the actual situation of Xiamen, the value is 17 days, which gives *γ* = *γ'* = 0.05882. The data for birth and death rates were obtained from the Xiamen Statistical Yearbook, and the annual birth and death rates for 2011 – 2020 range from 0.0100 to 0.0166 and 0.0029 to 0.0051, respectively. Since the simulation time in this study is in days, *μ* ranges from 2.74 × 10^–5^ to 4.55 × 10^–5^, *m* ranges from 7.95 × 10^–6^ to 1.40 × 10^–5^. The values of model parameters and their methods are shown in Table 1.

**Table 1:** Description and source of parameters in the SEIAR model.

Parameter	Description	Unit	Value	Range	Method
*β*	Transmission relative rate		–	≥0	Curve fitting
*κ*	Relative transmissibility rate of asymptomatic to symptomatic individuals	1	0.1	0–1	Reference (Chen et al., 2020)
*p*	Proportion of the asymptomatic	1	0.2	0.15–0.27	Reference (Wu et al., 2020)
*ω*	Incubation relative rate	days^-1^	0.08333	0.05–0.1	Reference (Chen et al., 2020)
*ω′*	Latent relative rate	days^-1^	0.08333	0.05–0.1	Reference (Chen et al., 2020)
*γ*	Removed rate of the infectious	days^-1^	0.05882	0.04–0.2	Reference (Pan et al., 2012)
*γ′*	Removed rate of the asymptomatic	days^-1^	0.05882	0.04–0.2	Reference (Pan et al., 2012)
*μ*	Natural birth rate	days^-1^	2.74 × 10^–5^ to 4.55 × 10^–5^	0–1	Xiamen Statistical Yearbook
*m*	Death rate	days^-1^	7.95 × 10^–6^ to 1.40 × 10^–5^	0–1	Xiamen Statistical Yearbook

### 2.5 Evaluation transmission

Previous studies have used the *basic reproduction number* (*R*_0_), the *effective reproduction number* (*R*_*eff*_), and the *time-dependent reproduction number* (*R*_*t*_) to evaluate the transmission (Stoddard et al.).

(1) the *basic reproduction number* (*R*_0_): The number of new cases expected to be generated during the infectious period of a case imported into a susceptible population without any intervention.
(2) the *effective reproduction number* (*R*_*eff*_): The number of new cases expected to be generated during the infectious period of one case introduced into the susceptible population when the population is not fully susceptible or when interventions are taken.
(3) the *time-dependent reproduction number* (*R*_*t*_): The average number of new cases that can result from one case of disease at time *t*.

In this paper, *R*_*t*_ was calculated by the next-generation matrix method to quantify the transmissibility of mumps.

When we apply the next-generation matrix method to attain *R*_*t*_, first, we need to divide the five compartments into two classes: the infected class, including (*E*, *A*, *I*), and the uninfected class, including (*S*, *R*). The increase in the infected class and the transfer among the infected class are what we are concerned about. *F* donates a new infection rate, while *V* indicates all other rates. Here:

$$F=\left(\begin{array}{l} \frac{\beta S(I+\kappa A)}{N}\\ 0\\ 0 \end{array}\right)$$

$$\text{V}=\left[\begin{array}{l} \omega E+mE\\ -\left(1-p\right)\omega E+\left(\gamma +m\right)I\\ -p\omega^{'}E+\left(\gamma^{'}+m\right)A \end{array}\right]$$

Let *X*_*0*_ =(N, 0,0,0,0) be the Disease-Free equilibrium of the system. And the Jacobian matrix of *F* and *V* can be illustrated as

$$F^{\text{′}}=\left(\begin{array}{lll} 0 & \beta & \beta \kappa \\ 0 & 0 & 0\\ 0 & 0 & 0 \end{array}\right)$$

$$V^{'}=\left(\begin{array}{lll} \omega +m & 0 & 0\\ -\left(1-p\right)\omega & \left(\gamma +m\right) & 0\\ -p\omega^{'} & 0 & \left(\gamma +m\right) \end{array}\right)$$

Therefore,

$$V{'}^{-1}=\left[\begin{array}{lll} \frac{1}{\omega +m} & 0 & 0\\ \frac{\omega \left(1-p\right)}{\left(\omega +m\right)\left(\gamma +m\right)} & \frac{1}{\gamma +m} & 0\\ \frac{\omega p}{\left(\omega +m\right)\left(\gamma +m\right)} & 0 & \frac{1}{\gamma +m} \end{array}\right]$$

$$F^{\text{′}}V\prime^{-1}=\left(\begin{array}{lll} X_{1} & * & *\\ 0 & 0 & 0\\ 0 & 0 & 0 \end{array}\right)$$

$$X_{1}=\frac{\beta \omega \left(1-p\right)+p\kappa }{\left(\omega +m\right)\left(\gamma +m\right)}$$

and *X*_1_ is the only eigenvalue of *F'V'*^*−1*^.

This means, adopting the next generation matrix method, *R*_*t*_ can be calculated by *X*_1_.

$$R_{t}=X_{1}=\frac{\beta \omega \left(1-p\right)+p\kappa }{\left(\omega +m\right)\left(\gamma +m\right)}$$

### 2.6 Simulation method and statistical analysis

The *R* Programming Language (developed by Ross Ihaka, Robert Gentleman of the University of Auckland, New Zealand) was used for model simulating. The differential equations were solved by using the deSolve package and the bbmle package was used to fit the model. Curves were calibrated using maximum likelihood estimation (MLE), and the coefficient of determination (*R*^2^) was used to evaluate the fitting effect.

## 3 Results

### 3.1 Epidemiological characteristics of mumps

**Fig. 2:**
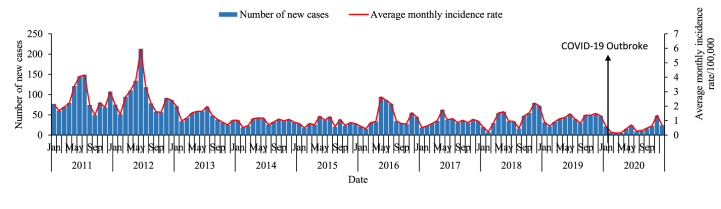
The epidemiological characteristics of mumps in Xiamen, 2011–2020.

A total of 5,831 cases of mumps have been reported in Xiamen from January 2011 to December 2020, with an average annual incidence of 15.111 per 100,000 people. No deaths have been reported. From 2011 to 2020, the overall incidence of mumps showed a decreasing trend from 2011 to 2020 (Fig. 2). The incidence rate increased from 30.166 per 100,000 people in 2011 to 32.016 per 100,000 people in 2012, then decreased year by year. There was a certain rebound and upward trend in 2016, followed by a decline to a low of 4.183 per 100,000 people in 2020. The number of new cases in 2020 declined significantly after the COVID-19 outbreak, which decreased from 1,089 in 2011 to 216 in 2020, a decrease of 80.17%.

### 3.2 Curve fitting results

**Fig. 3:**
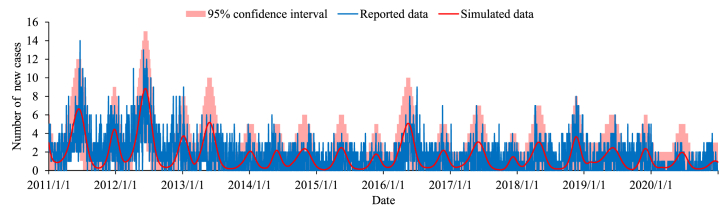
The results of model fitting.

The model fitted the reported data well (*R*^2^ = 0.246, *P* < 0.001). The results of model fitting and the cases of mumps were shown in Fig. 3. The model-fitting results showed that there were 5,651 (95% Confidence interval: 480 to 14,959) cases of mumps from 2011 to 2020, with a difference of 2.72% from the actual data. Meanwhile, in supplementary material, we performed sensitivity analysis for the model parameters. The results showed that the parameters *κ,* *p, ω and ω′* have little effect on the model results, while *γ* and *γ'* affected the model results (Figure A and Figure B). Because we used seasonal parameters in the model, the cumulative number of cases did not increase or decrease monotonically when gradually increasing a parameter.

### 3.3 Transmissibility of mumps

**Fig. 4:**
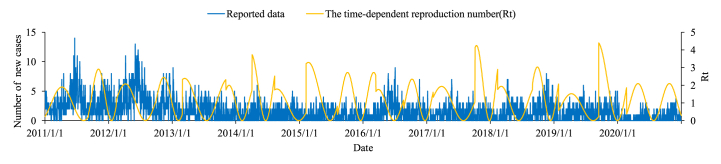
The number of new cases of mumps and *R*_*t*_ in Xiamen.

The results of the number of new cases showed that the prevalence of mumps in Xiamen was seasonal (Fig. 4), with a significant increase in the number of new cases in May – July and November – January, showing a bimodal epidemic. The *R*_*t*_ lasted at the median of 1.091 (0 – 4.393). The peak times of transmissibility were February – April and September – November respectively.

### 3.4 Meteorological factors

**Fig. 5:**
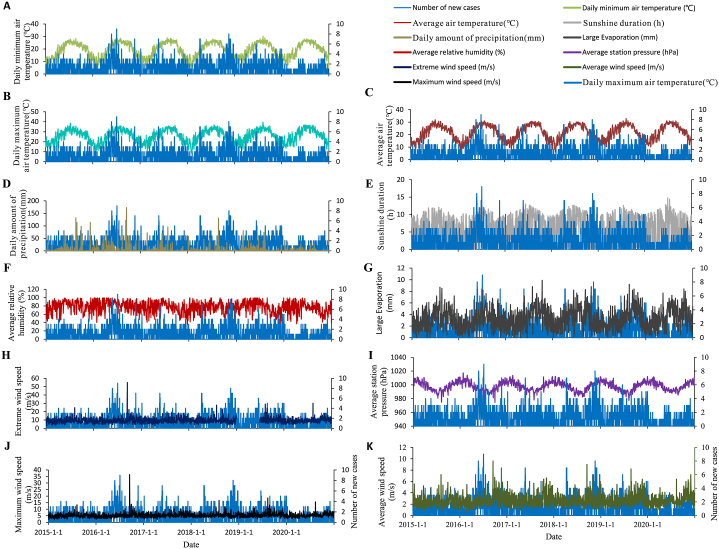
The time-series distribution of daily mumps cases and meteorological variables in Xiamen, 2015–2020.

**Fig. 6:**
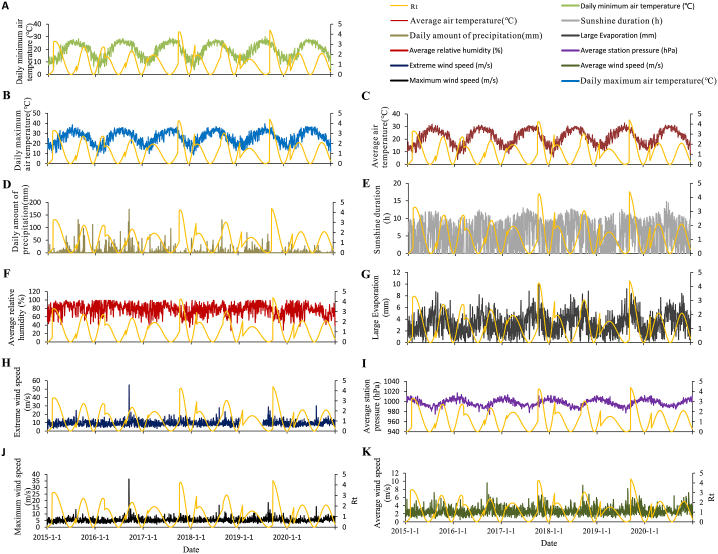
The time-series distribution of *R*_t_ and meteorological variables in Xiamen, 2015–2020.

From 2015 to 2020, meteorological events in Xiamen indicate a clear seasonality (Fig. 5 and Fig. 6). The temperatures show a unimodal distribution, with the highest temperature concentrated in July and August. The longest hours of sunshine are from June to August as well. Daily amount of precipitation present a bimodal distribution, mostly in March to May and August to September. The peak of significant evaporation is in July – October. A periodicity of the average relative humidity could be observed, with the humidity remaining at a high level from June to September. The average air pressure at this station is cyclical, peaking from December to January of the following year. The seasonality of the wind speed is less obvious and it has a larger value in August, September, and November.

### 3.5 The correlation between mumps and meteorological factors

**Fig. 7:**
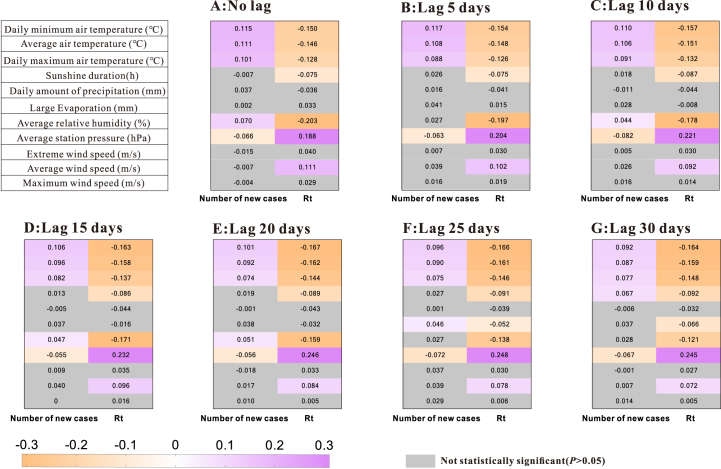
Pearson correlation analysis between mumps and meteorology.

As shown in Fig. 7, Pearson correlation analysis of non-lag meteorological factors and the number of cases showed that temperature (daily minimum air temperature (Pearson correlation coefficient [*r*] = 0.115), average temperature (*r* = 0.111), daily maximum temperature (*r* = 0.101)), average relative humidity (*r* = 0.070) was positively correlated with the number of new cases, while average station pressure (*r* = -0.066) was negatively correlated with the number of cases.

The Pearson correlation analysis of non-lagged meteorological factors and the mumps transmissibility showed that the average station pressure (*r* = 0.188) and the average wind speed (*r* = 0.111) were positively correlated with the transmissibility, in contrast to air temperature (daily minimum air temperature (*r* = -0.150), average air temperature (*r* = -0.146), daily maximum air temperature (*r* = -0.128)), sunshine duration (*r* = -0.075) and average relative humidity (*r* = -0.203) (Fig. 7).

**Fig. 8:**
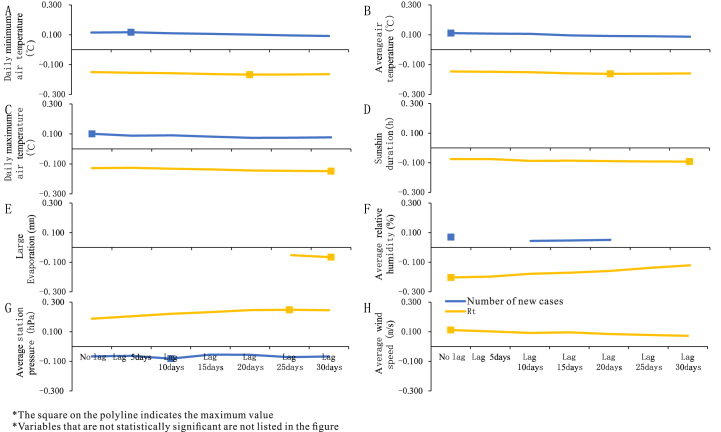
Lag sequence diagram of Pearson correlation analysis between meteorology and mumps.

When it gradually lagged different days, daily minimum air temperature (*r* ranged: 0.092 to 0.117), average air temperature (*r* ranged: 0.087 to 0.111), daily maximum air temperature (*r* ranged: 0.074 to 0.101), average relative humidity (*r* ranged: 0.044 to 0.070), average station pressure (*r* ranged: -0.055 to -0.082) had a continuous effect on the number of new cases (Fig. 8).

When the meteorological variables were lagged by different days, transmittance was correlated with average station pressure (*r* ranged: 0.188 to 0.248), average wind speed (*r* ranged: 0.072 to 0.111), daily minimum air temperature (*r* ranged: -0.150 to -0.167), average air temperature (*r* ranged: -0.146 to -0.162), daily maximum air temperature (*r* ranged: -0.126 to -0.148), sunshine duration (*r* ranged: -0.075 to -0.092), average relative humidity (*r* ranged: -0.121 to -0.203) (Fig. 8).

## 4 Discussion

### 4.1 Description of mumps

As far as we know, this is the first time that Xiamen has used the data of mumps cases from 2011 to 2020 to analyze the correlation between mumps and meteorological monitoring data to provide a certain basis for the prevention and control of mumps.

In Fujian Province, the proportion of cases in the two peak cases of mumps has changed in recent years (Li et al., 2018). In our study, the pattern of epidemiological curve of mumps cases in Xiamen showed some changes from 2011 to 2020. During the 10 years, the cases of mumps in Xiamen showed an overall decreasing trend. From the time of onset, the number of cases of mumps in Xiamen showed a clear seasonal bimodal distribution. From 2011 to 2017, the main peak occurs in May to July and the secondary peak occurs in November to December. From 2018 to 2020, the main peak is in November, and the secondary peak is from May to June. From 2011 to 2020, the first peak decreased significantly, and the secondary peak decreased slowly. In terms of the number of cases in the peak period as a percentage of the year, the proportion in the first peak drops from 15.18% in 2011 to 11.63% in 2020, and the proportion in the second peak period increases from 9.28% in 2011 to 21.86% in 2020. The peak occurs after the start of the school year, which is different from the time coinciding with the start of school reported by Li et al.(Li et al., 2018; Liu et al., 2015; Su et al., 2016). One reason may be due to geographical differences. Another possibility is that these studies ignore the natural history of the disease, skipping the process of the disease transmission, and focusing only on the outcome of the disease. On this basis, the transmissibility of the disease is analyzed in this paper, and the specific analysis is elaborated in the next paragraph. At present, the different decreasing ranges of the two peaks and the reasons for the alternation of primary and secondary peaks are still unclear, which require further monitoring and analysis. It is worth noting that the first peak from 2014 to 2018 occurs in May, and the first peak from 2019 to 2020 shows a certain backward shift and occurs in June.

### 4.2 Transmissibility

The results of the time-dependent reproduction number indicated that the transmissibility of the mumps in Xiamen showed an obvious seasonality. The *R*_*t*_ of mumps ranged from 0 to 4.393, and the peak periods of mumps transmissibility were from February to April and from September to November, which was consistent with the start of the school year. Generally speaking, the real-time transmission ability of the mumps can be evaluated by the time-dependent reproduction number (Stoddard et al.). When *R*_*t*_ > 1, the disease continues to spread, when *R*_*t*_ = 1, the epidemic will not spread or stop, when *R*_*t*_ < 1, the epidemic tends to stop. We found that the duration of continuous spread (*R*_*t*_ > 1) was mainly from February to May and from September to November. In contrast, the duration of *R*_*t*_ < 1 was mainly from July to August, and from December to January. Also, since the incubation period of mumps is 12 – 28 days, when students return to school, mumps started to spread in the population, and cases occur after approximately one incubation period, which coincides with the peak in the number of new cases mentioned above. This explains well the higher incidence of cases during the school period and the lower cases during the school-leaving period. Therefore, the key time for prevention and control measures in Xiamen City should be at least one incubation period ahead of the onset of cases, that is, during the months of peak transmission for focused prevention and control.

### 4.3 Meteorological factors and mumps

Several studies have shown a nonlinear relationship between temperature and the mumps cases (Hu et al., 2018, Zhang, Zhou, et al., 2019). A time-series study showed that both high and low temperatures increase the risk of disease (Yu, Yang, Wei, et al., 2018). For air temperature, the risk is higher in cold weather than in hot weather. This is consistent with the findings of Hu(Hu et al., 2018). Also, Hu suggested that the incidence of mumps increases when the temperature is higher than 20 °C. Meteorology is a factor that influences the development of mumps. However, the main factors influencing the development of mumps are the virus and the host. In our article, the association between meteorological factors and mumps was not very strong, with the strongest correlation coefficient between the two not exceeding 0.25. The results of other studies showed that the strength of the association between meteorology and mumps was similar to the results of this paper. In this study, temperature was positively correlated with the mumps cases and negatively correlated with the transmission capacity. The biological characteristics of the mumps virus indicate that the virus has relatively low resistance to heat and can be died at 55 °C in 30 min. Therefore, it is believed that higher temperature has an impact on the biological activity of the mumps virus, thereby reducing the transmissibility. Nevertheless, higher temperatures also affect human activities, and increase contact with people, which in turn increases the number of cases. In addition, the longer the daylight hours, the higher the temperature, thus also explaining the negative correlation between sunlight duration and transmissibility.

Higher average relative humidity increased the number of mumps cases, which is consistent with the studies of Hu et al.(Hu et al., 2018; Lu et al., 2020; Onozuka & Hashizume, 2011; Yang et al., 2014), but contrary to the results of Li et al.(Li et al., 2017, Zhang, Zhou, et al., 2019). Studies have shown that people tend to stay outdoors in crowded areas on days with high relative humidity in Guangzhou, which increases the risk of illness (Shaman & Kohn, 2009). Differences in results may be due to differences in analytical methods. On the other hand, higher average relative humidity reduced the ability of the mumps virus to spread. Low relative humidity causes water in virus-carrying droplets to evaporate faster, settle more slowly and remain in the air longer, increasing the chance of inhalation in susceptible individuals (Lowen et al., 2007; Richard & Fouchier, 2016).

In this study, atmospheric pressure was negatively correlated with the number of new cases, which is consistent with the study in Beijing (Hao et al., 2019), but other studies showed no correlation (Yu, Yang, Yu, et al., 2018). Researches on the influence mechanism of atmospheric pressure are limited. We believe that changes in atmospheric pressure are usually associated with rainfall and wind speed, which may affect people's outdoor activities, as well as human contact. In addition, this study found a positive correlation between air pressure, mean wind speed and the transmissibility of mumps.

Also, we analyzed the impact of meteorological factors with different lag days on the number of mumps cases and *R*_*t*_. It was worth noting that not all the relevant meteorological factors mentioned above were statistically related, and only some variables were statistically significant. In the correlation between lagged meteorological variables and the number of cases, there was no obvious change in air temperature and average station pressure. The range of *r* between lagged average station pressure and transmissibility was greater than other meteorological variables. The effect of meteorological factors on the number of cases and *R*_*t*_ had a duration, which may be related to the incubation period of mumps. Meteorological factors could predict the onset of mumps to a certain extent (Zhang, Zhou, et al., 2019).

## 5 Conclusion

From 2011 to 2020, the epidemic intensity of mumps in Xiamen City showed a downward trend, with altering the distribution of primary and secondary peaks. However, relatively high transmissibility in Xiamen City has led to successive outbreaks of mumps. The obvious seasonality of mumps cases was mainly in May to July and November to December, and transmissibility was mainly in February to April and September to November. The peak time difference between the transmissibility and the number of cases suggests that the timing of prevention and control should be based on the peak transmissibility. Meteorological factors, especially air temperature and relative humidity, might be more strongly correlated with mumps than other factors and have a persistent effect over a certain period of time to predict the incidence of mumps. Therefore, the government can also improve prevention and control measures based on meteorology.

## Ethics approval and consent to participate

This effort of disease control was part of the CDC's routine responsibility in Xiamen City, Fujian Province, China. Therefore, institutional review and informed consent were not required for this study. All data analyzed were anonymous.

## Consent for publication

Not applicable.

## Availability of data and materials

All relevant data are within the paper and its Supporting Information.

## Funding

This study was partly supported by the 10.13039/100000865Bill & Melinda Gates Foundation (Grant INV-005834 to T.C.).

## Authors’ contributions

TMC, MZW, and QL designed research; JFH, ZYZ, WQL, PHL, ZYL, LL, CL, and TMC collected data; JFH, ZYZ, MZW, and WQL wrote the original draft; JFH, ZYZ, JR, BD, WKL, TLY, YFW, TMC, MZW, and BZ were responsible for data curating; JFH, ZYZ, and WQL analyzed the data. JFH, ZYZ, QL, MZW, and TMC reviewed and edited the manuscript. All authors read and approved the final manuscript.

## Declaration of competing interest

The authors declare that they have no known competing financial interests or personal relationships that could have appeared to influence the work reported in this paper.

## References

[bib1] Chen T.M., Rui J., Wang Q.P., Zhao Z.Y., Cui J.A., Yin L. (2020). A mathematical model for simulating the phase-based transmissibility of a novel coronavirus. Infectious Diseases of Poverty.

[bib2] Chernov A.A., Kelbert M.Y., Shemendyuk A.A. (2020). Optimal vaccine allocation during the mumps outbreak in two SIR centres. Mathematical Medicine and Biology: A Journal of the IMA.

[bib3] Court B.M.S. (2006). Law of the People's Republic of China on the prevention and control of infectious diseases. Chinese Law and Government.

[bib4] Czumbel I., Quinten C., Lopalco P., Semenza J.C. (2018). Management and control of communicable diseases in schools and other child care settings: Systematic review on the incubation period and period of infectiousness. BMC Infectious Diseases.

[bib5] Hao Y., Wang R.R., Han L., Wang H., Zhang X., Tang Q.L. (2019). Time series analysis of mumps and meteorological factors in Beijing, China. BMC Infectious Diseases.

[bib6] Hu W., Li Y., Han W., Xue L., Zhang W., Ma W. (2018). Meteorological factors and the incidence of mumps in Fujian Province, China, 2005-2013: Non-linear effects. The Science of the Total Environment.

[bib7] Hviid A., Rubin S., Muhlemann K. (2008). Mumps. Lancet.

[bib8] Jiang R.J., Yin Q.Z., Xu M.J., Zhao Z.M., Deng Y., Che Y.C. (2019). [Epidemiological characteristics of mumps in mainland China from 2004 to 2018 and key population for prevention and control]. Zhong Guo Dang Dai Er Ke Za Zhi.

[bib10] Li D., Chen Z.F., Yang X.H., Pan W.Y., Wang Q., Zhang S.H. (2018). [Epidemiological and pathogenic characteristics of mumps in Fujian province, 2005-2017]. Zhonghua Liuxingbingxue Zazhi.

[bib11] Li Y., Liu X., Wang L. (2017). Modelling the transmission dynamics and control of mumps in mainland China. International Journal of Environmental Research and Public Health.

[bib12] Liu D., Meng L., Gou F., Wei K., Yang X., Liu X. (2015). [Spatial temporal distribution of mumps in Gansu, 2009-2013]. Zhonghua Liuxingbingxue Zazhi.

[bib13] Lowen A.C., Mubareka S., Steel J., Palese P. (2007). Influenza virus transmission is dependent on relative humidity and temperature. PLoS Pathogens.

[bib14] Lu J., Yang Z., Ma X., Ma M., Zhang Z. (2020). The role of meteorological factors on mumps incidence among children in Guangzhou, Southern China. PLoS One.

[bib15] Onozuka D., Hashizume M. (2011). Effect of weather variability on the incidence of mumps in children: A time-series analysis. Epidemiology and Infection.

[bib9] Pan J.R., Huang Z.Q., Chen K. (2012). Evaluation of outbreak control effect of varicella by time-delay discrete SEIR model in journal of mathematical medicine. Chinese Journal of Preventive Medicine.

[bib16] Qu Q., Fang C., Zhang L., Jia W., Weng J., Li Y. (2017). A mumps model with seasonality in China. Infectious Disease Modelling.

[bib17] Richard M., Fouchier R.A. (2016). Influenza A virus transmission via respiratory aerosols or droplets as it relates to pandemic potential. FEMS Microbiology Reviews.

[bib18] Shaman J., Kohn M. (2009). Absolute humidity modulates influenza survival, transmission, and seasonality. Proceedings of the National Academy of Sciences of the United States of America.

[bib19] Stoddard S.T., Forshey B.M., Morrison A.C., Paz-Soldan V.A., Vazquez-Prokopec G.M., Astete H. (2013). House-to-house human movement drives dengue virus transmission. Proceedings of the National Academy of Sciences.

[bib20] Su Q.R., Liu J., Ma C., Fan C.X., Wen N., Luo H.M. (2016). [Epidemic profile of mumps in China during 2004-2013]. Zhonghua Yufang Yixue Zazhi.

[bib21] Wu H., You E., Jiang C., Yang Y., Wang L., Niu Q. (2020). Effects of extreme meteorological factors on daily mumps cases in Hefei, China, during 2011-2016. Environmental Science and Pollution Research International.

[bib22] Yang Q., Yang Z., Ding H., Zhang X., Dong Z., Hu W. (2014). The relationship between meteorological factors and mumps incidence in Guangzhou, China, 2005-2012. Human Vaccines & Immunotherapeutics.

[bib23] Yu G., Yang R., Wei Y., Yu D., Zhai W., Cai J. (2018). Spatial, temporal, and spatiotemporal analysis of mumps in Guangxi Province, China, 2005-2016. BMC Infectious Diseases.

[bib24] Yu G., Yang R., Yu D., Cai J., Tang J., Zhai W. (2018). Impact of meteorological factors on mumps and potential effect modifiers: An analysis of 10 cities in Guangxi, Southern China. Environmental Research.

[bib25] Zha W.T., Li W.T., Zhou N., Zhu J.J., Feng R., Li T. (2020). Effects of meteorological factors on the incidence of mumps and models for prediction, China. BMC Infectious Diseases.

[bib26] Zhang D., Guo Y., Rutherford S., Qi C., Wang X., Wang P. (2019). The relationship between meteorological factors and mumps based on Boosted regression tree model. The Science of the Total Environment.

[bib27] Zhang Q., Zhou M., Yang Y., You E., Wu J., Zhang W. (2019). Short-term effects of extreme meteorological factors on childhood hand, foot, and mouth disease reinfection in Hefei, China: A distributed lag non-linear analysis. The Science of the Total Environment.

