# Supplementary figures and images for: Correlation between mumps and meteorological factors in Xiamen City, China: A modelling study
Source: Infect Dis Model. 2022 Apr 24;7(2):127–37. doi: 10.1016/j.idm.2022.04.004 (PMC9062423; doi:10.1016/j.idm.2022.04.004)

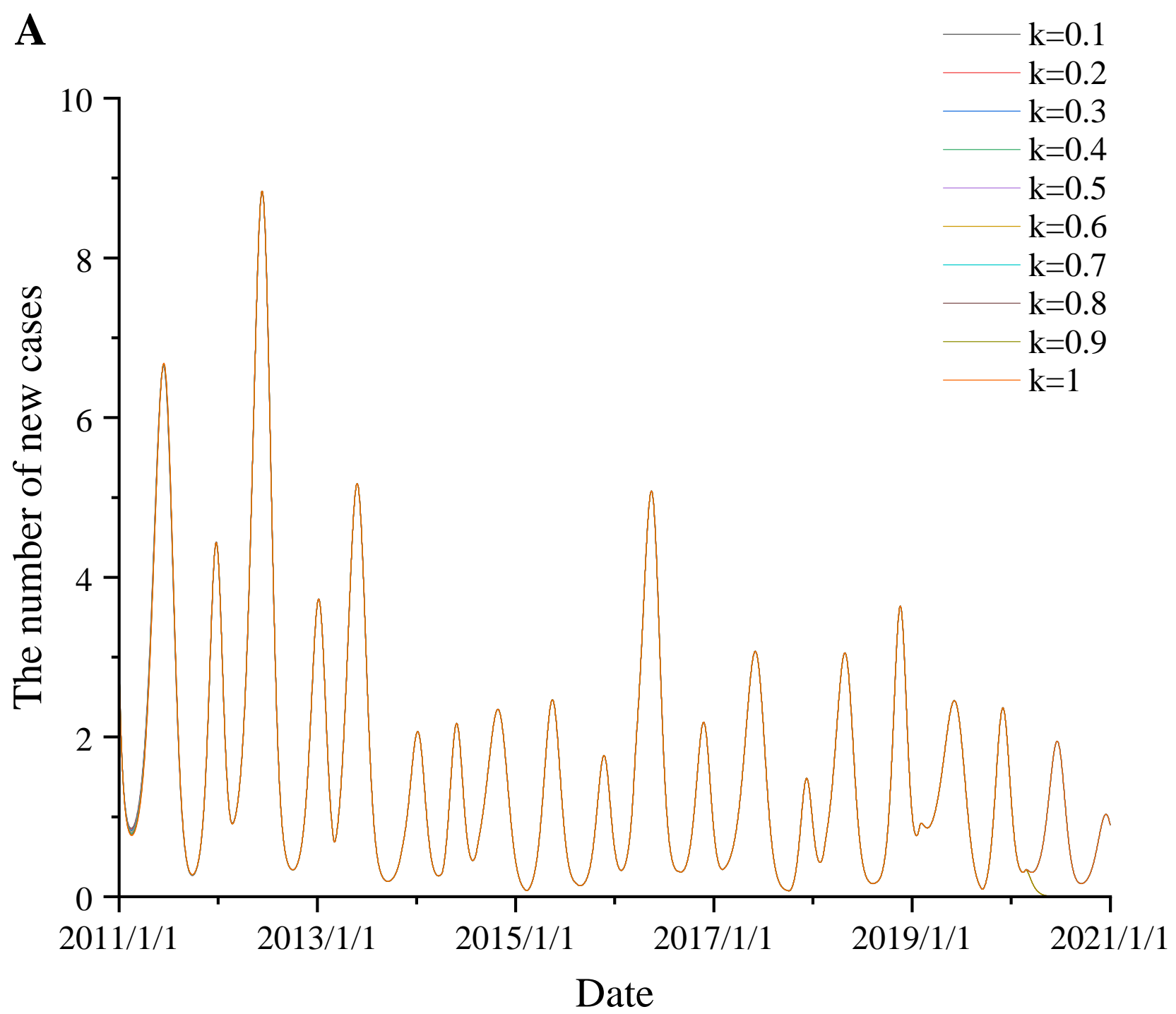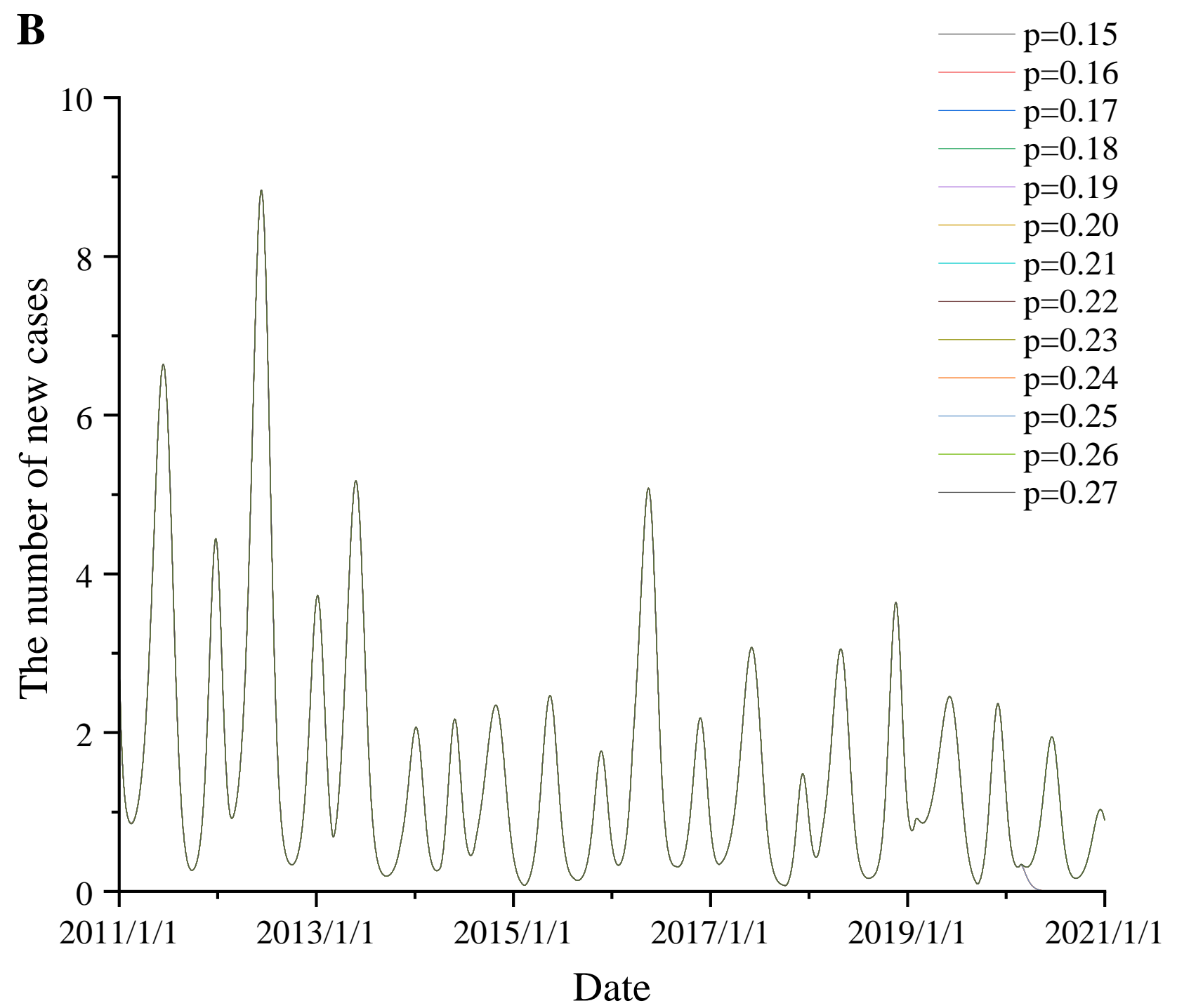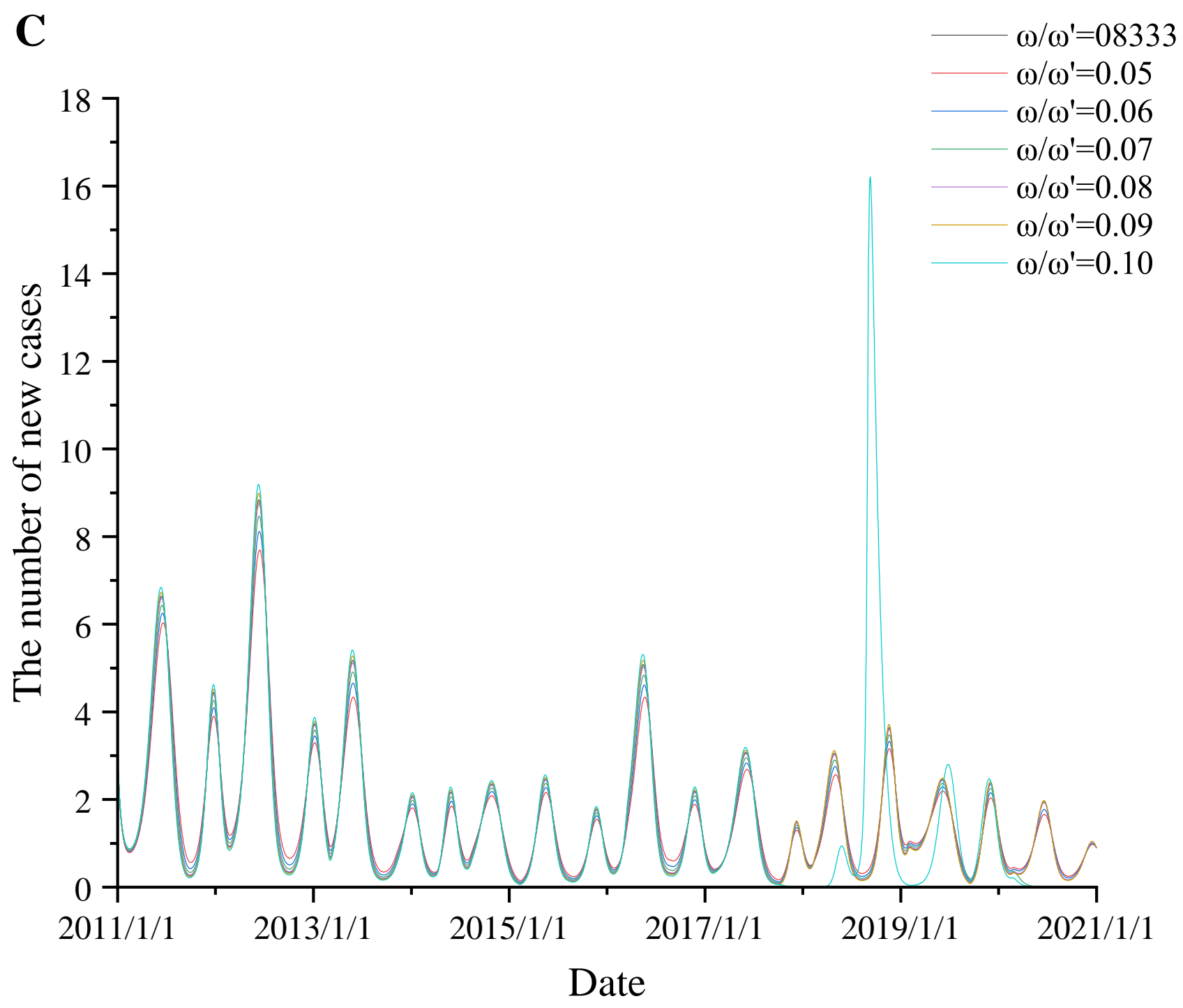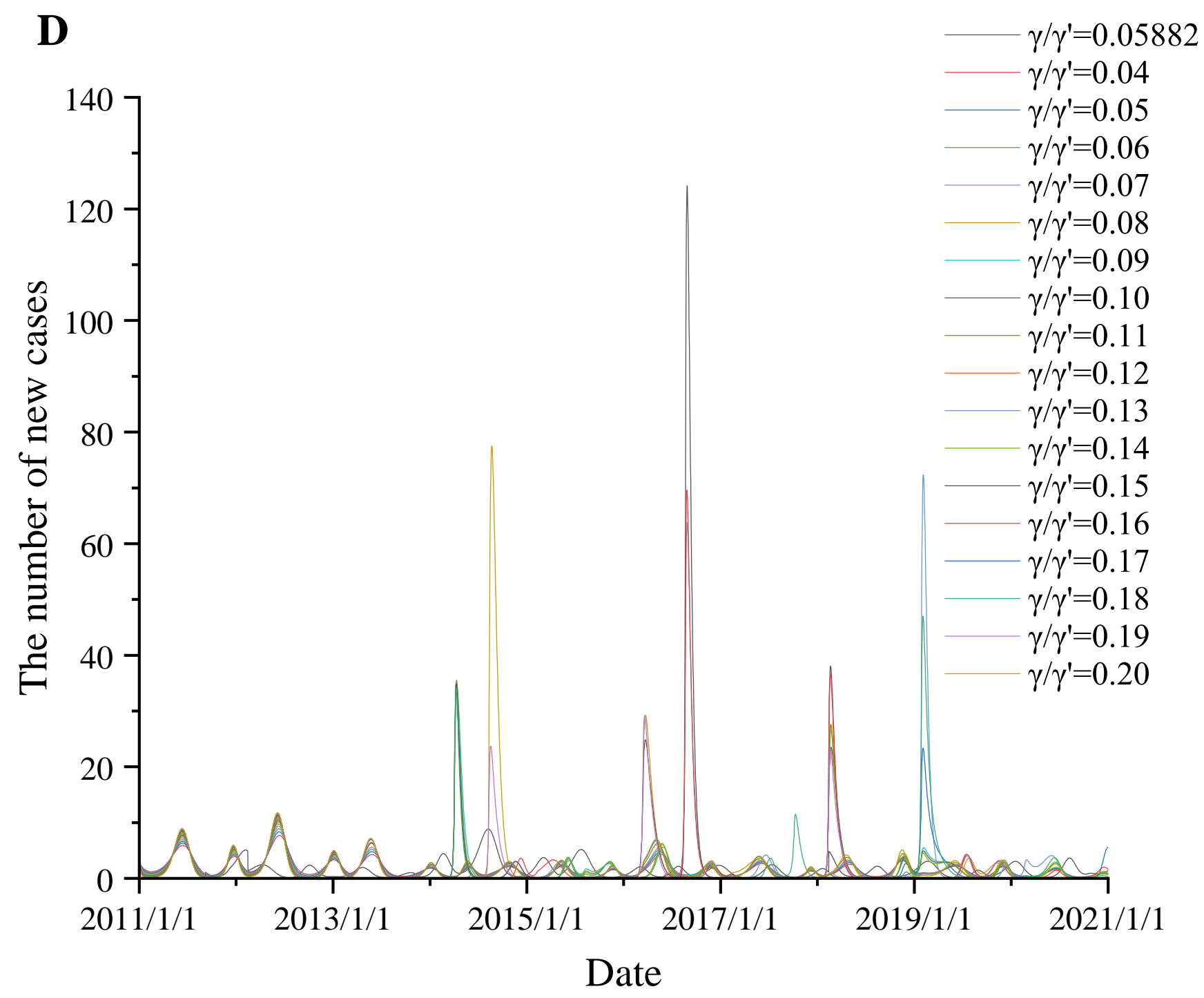

Supplement: Multimedia component 1 [file mmc1.pdf]

A

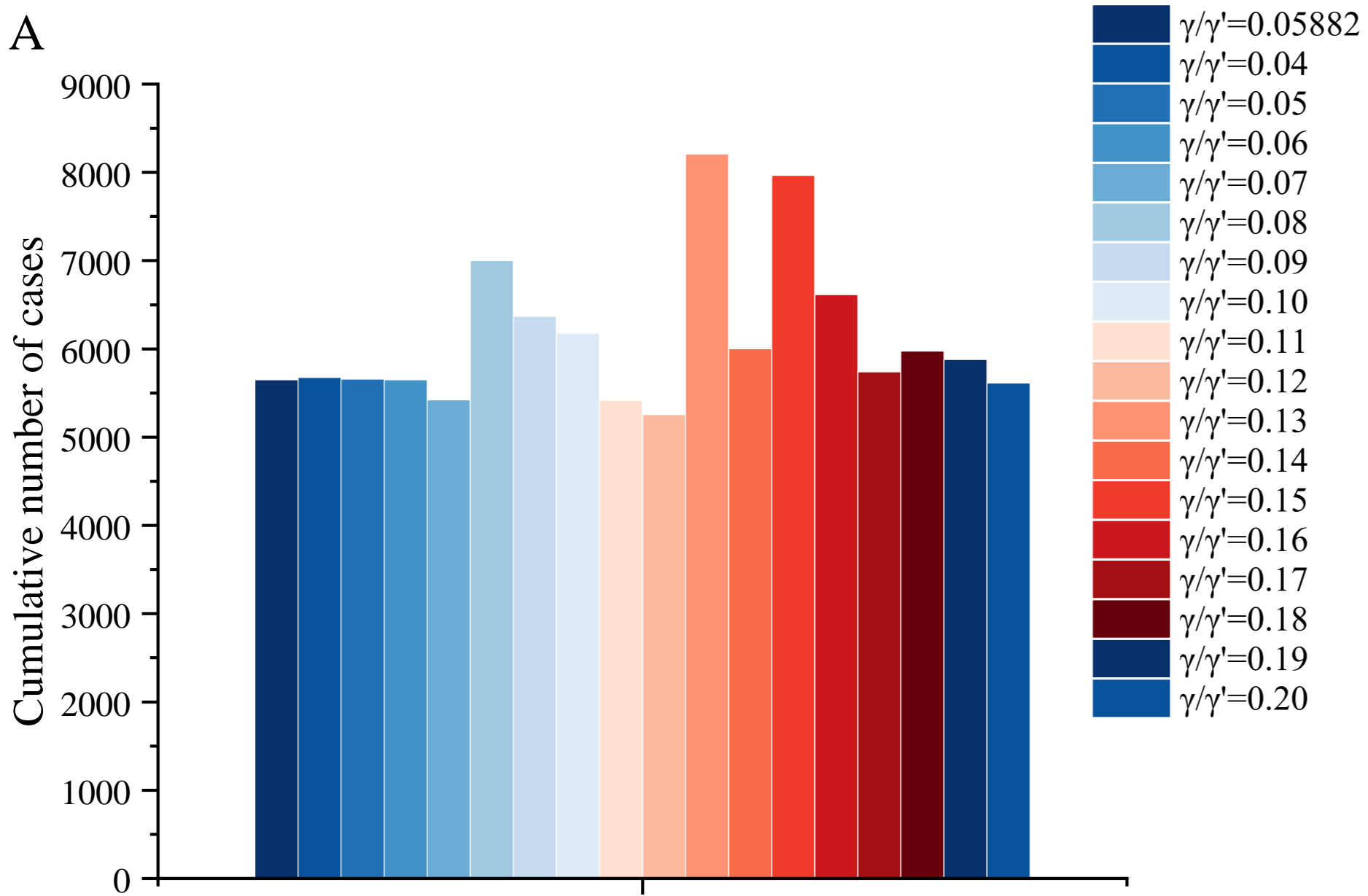

B

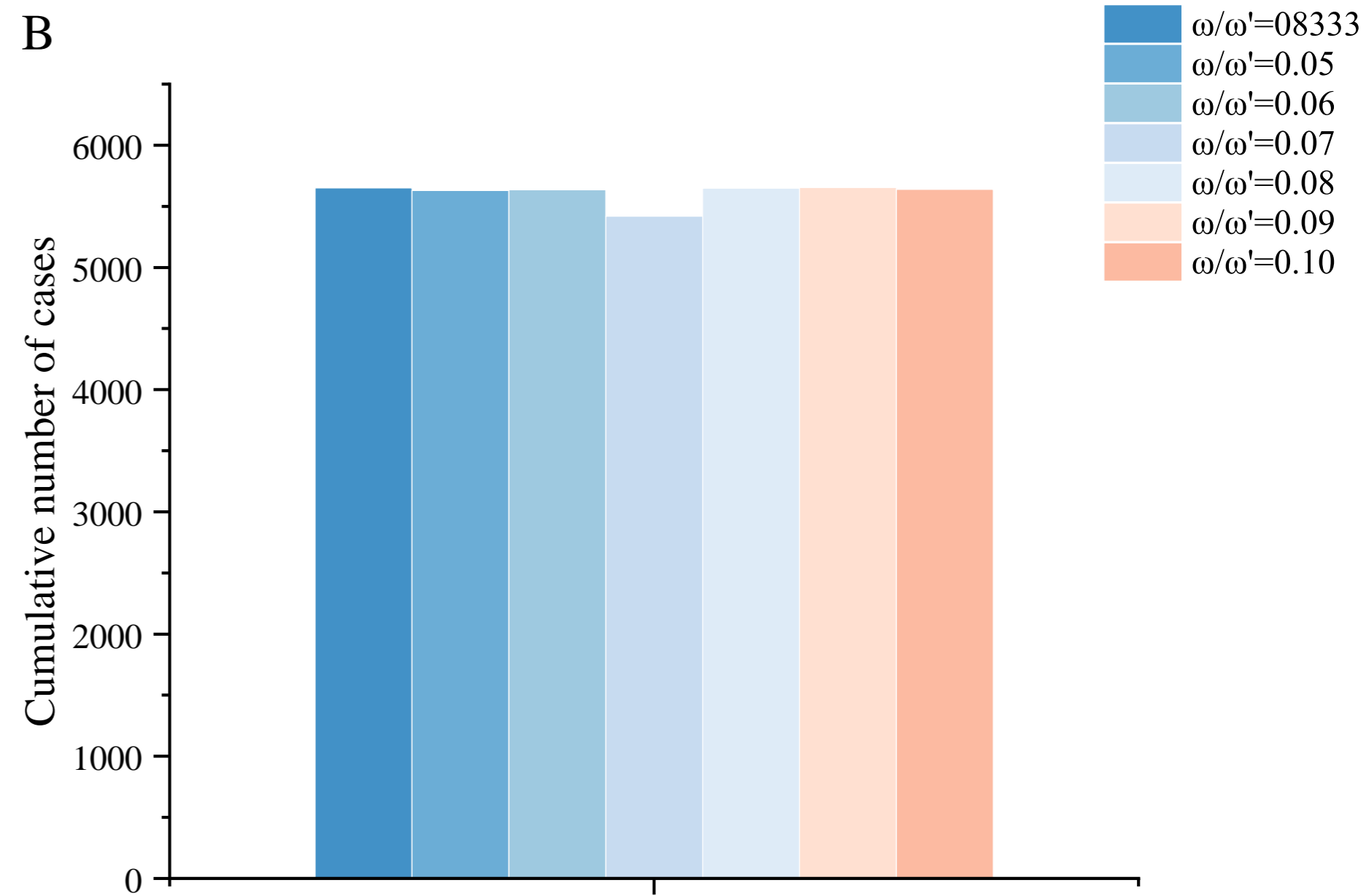

C

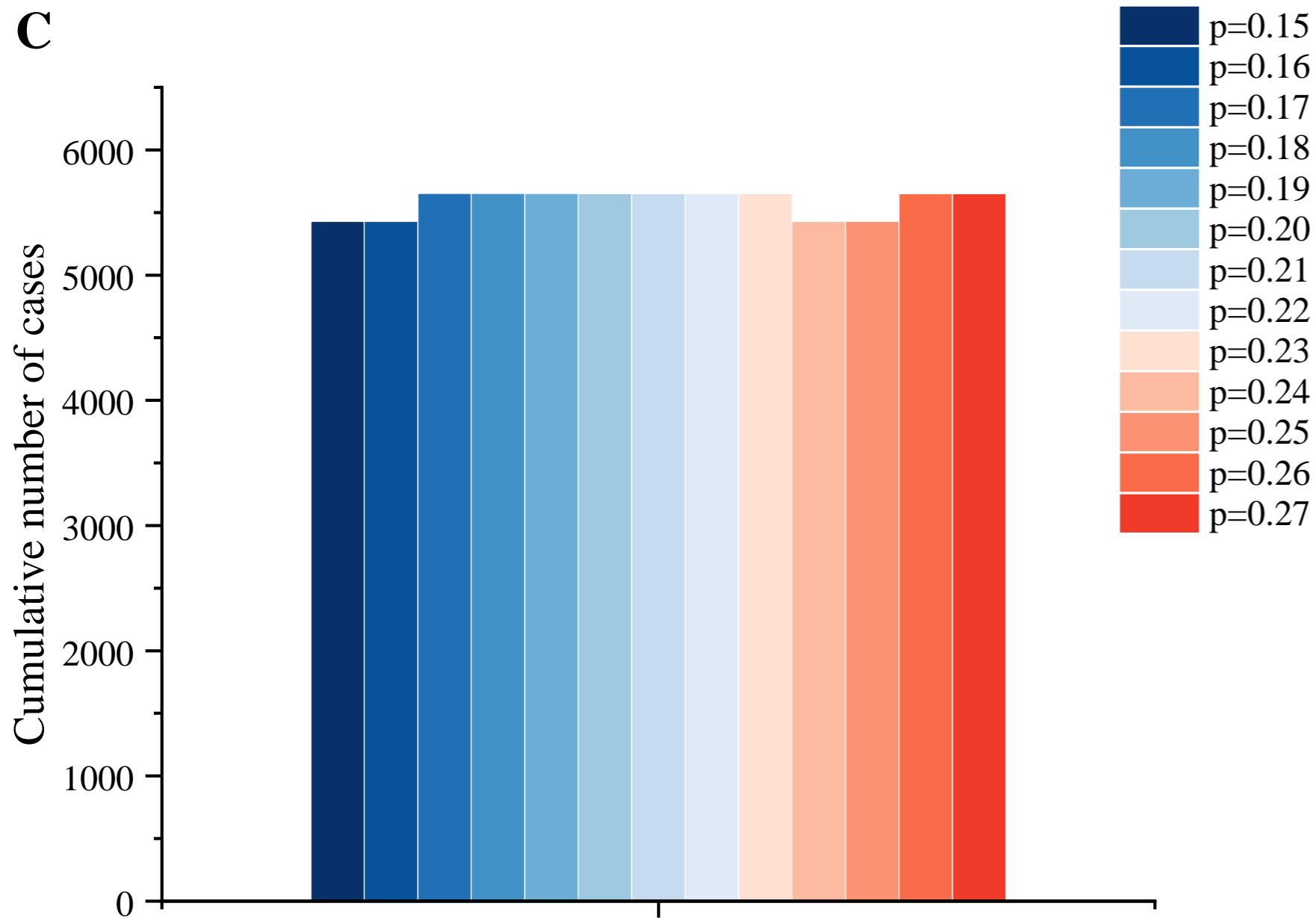

D

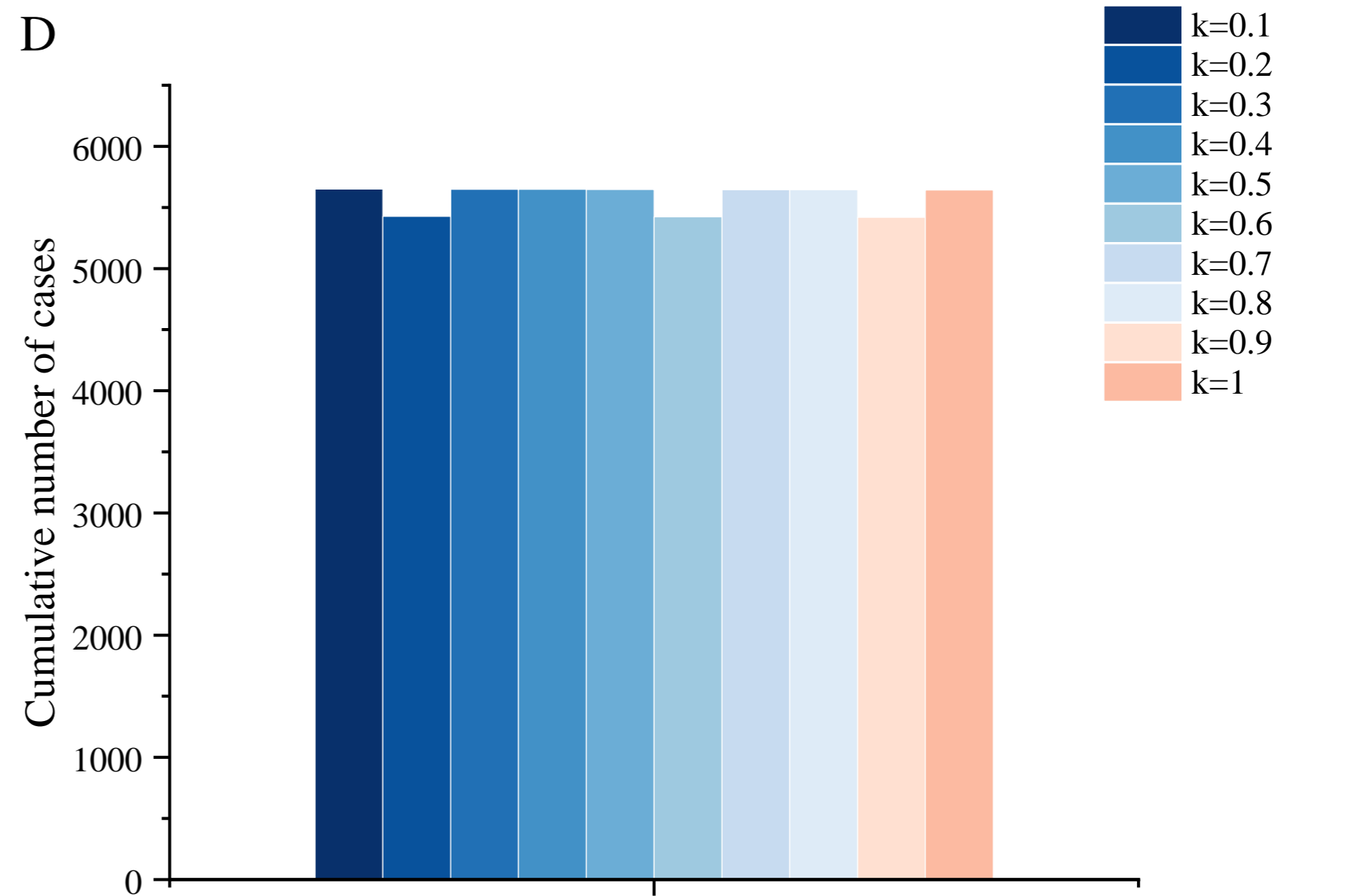

Sensitivity analyses

Supplement: Multimedia component 2 [file mmc2.pdf]
